# The relationship of stress level and periodontal tissue health in soldiers from Kodam Jaya: a cross-sectional study

**DOI:** 10.3389/froh.2025.1574112

**Published:** 2025-08-15

**Authors:** Baby Prabowo Setyawati, Nadila Dita, Ishiko Herianto, Ernie Maduratna Setiawatie

**Affiliations:** ^1^ Dental and Oral Health Institution of Army Health Center (Lakesgilut Puskesad), Jakarta, Indonesia; ^2^ Gatot Soebroto Army Hospital, Jakarta, Indonesia; ^3^ Departement of Periodontia, Faculty of Dentistry, Airlangga University, Surabaya, Indonesia

**Keywords:** stress, stress level, periodontal, periodontal tissue health, soldier, BoP examination

## Abstract

**Background:**

Stress plays a crucial role in the development of periodontal diseases, along with other behavioral factors. Professions with high-stress levels, like the Indonesian National Army, face challenges due to demanding requirements on skills, physical endurance, and mental strength. This study aims to explore the oral and periodontal health of soldiers, with a focus on the relationship between stress levels and periodontal conditions in soldiers from Kodam Jaya.

**Methods:**

The research method is observational with a cross-sectional approach involving 48 male respondents selected using a purposive sampling technique. All participants completed ten closed-ended questionnaires about emotions and thoughts over the past month, along with the Perceived Stress Scale (PSS). Data processing was analyzed using univariate and bivariate methods, including Spearman's correlation.

**Results:**

The analysis of stress levels among soldiers shows that the light category includes 11 individuals (22.9%), the moderate category consists of 29 individuals (60.4%), and the heavy category includes 8 individuals (16.7%). Therefore, out of the total 48 responses, the moderate stress level is the most common among soldiers. In terms of the distribution of BoP examinations, 7 cases (14.6%) met the healthy criteria, 2 cases (4.2%) met the mild criteria, 5 cases (10.4%) met the moderate criteria, and 34 cases (70.8%) met the severe criteria. The correlation between stress level and periodontal tissue health at BoP examination shows a moderate relationship with an r-value of 0.407 and a *p*-value of 0.004, indicating a significant relationship.

**Conclusion:**

There is a notable link between stress and periodontal tissue health, along with the Bleeding on Probing (BoP) assessment, in soldiers from Kodam Jaya.

## 1 Introduction

According to the World Health Organization (WHO), estimated that over 450 million individuals globally are impacted by high levels of stress. The WHO believes that one in four individuals will, at some stage in their lives, fulfill the criteria for a mental disorder. Over 350 million individuals globally are impacted by depression, rendering it one of the most prevalent mental disorders. The data revealed a higher incidence of stress among women (54.62%) compared to men (45.38%) (1). Stress is a consequence of the body's inability to adjust to a constantly changing environment, which can have both positive and negative effects on physical and emotional well-being (2).

The Indonesian National Army (TNI) is a state institution responsible for defending and protecting the country's sovereignty and integrity. Their duties require constant vigilance, operating 24/7 throughout the year. To maintain their professionalism and effectiveness, it is essential for TNI members to take care of both their physical and mental health (3).

Burnout syndrome may be induced or intensified by occupational factors, making early diagnosis and the implementation of preventive strategies essential. This is a widely recognized reality in contemporary times. Burnout syndrome is estimated to affect around 8% of the German labor force. A study involving 7,400 Czech physicians revealed that 34% perceive themselves as experiencing symptoms of tiredness, while 83% consider themselves at risk of getting it (4). Similarly, Indonesia faces a substantial burden of work stress, as shown by the Basic Health Research (Riskesdas) conducted by the Ministry of Health, indicating a mental and emotional disorder rate of 9.8, with over 35% of cases being severe and an estimated 43% resulting in lost work days (5).

There is the potential for stress in all jobs. On the other hand, the military is one of the jobs that can cause a lot of stress. Because they are so hard, military deployments are often the subject of study on stress. Support missions, peacekeeping efforts, or combat activities are just a few of the many ways that deployment can be shown. In and of itself, a combat action in Afghanistan is harder, especially when it comes to traumatic stress, than a logistical assignment in a third country with less danger. Whatever the position of deployed troops is, daily pressures will still show up. Optimal soldier health is required to enable the execution of tasks as one of the main components of national defense (6). Stress occurs in an individual and has many negative effects on the body. One of them is on dental and oral health (7). Optimal periodontal health is essential for general well-being. This includes the existence of healthy gums, fresh breath, intact periodontal tissue, and the lack of plaque or tartar buildup (8).

The likelihood of developing periodontal disease can be increased by a variety of factors. Stress factors are also of great importance, in addition to age, gender, education, socioeconomic status, exercise frequency, smoking habits, genetic conditions such as hypertension, and adherence to appropriate oral hygiene (9). The oral cavity is fundamentally connected to the health of periodontal tissue, facilitating consumption, speech, and communication without functional impairments, preserving cosmetic integrity, and preventing discomfort associated with disease. The periodontal tissue, consisting of the gingiva, alveolar bone, periodontal ligament, and cementum, is attached to the bone in a way that makes separation from the cavity unfeasible (10).

Periodontal disease is a result of chronic inflammation of tissues surrounding the teeth due to an imbalance between dental and oral hygiene and the body's response (11). In the Serbian armed forces, 84% of individuals needed motivation to uphold dental and oral health through scaling and root planing, while 41.2% required complex periodontal treatment. Only 3% of the total Serbian Armed Forced (SAF) population were identified as having a completely healthy periodontium, accounting for 48 individuals out of 1,411 (12).

Army Dikjurtakes Abit Dikmata soldiers with a periodontal health status score of 2 reached 60.78%. Out of the total, 31 individuals had subgingival calculus, representing 17.65% of the group. Additionally, 9 individuals, accounting for 11.76%, fell into the category of no bleeding, tartar, shallow pockets, and deep pockets with a score of 0, indicating healthy periodontal tissue. Furthermore, 6 individuals, making up 9.80% of the soldiers, exhibited a score of 3, indicating shallow pockets. Interestingly, none of the soldiers had a score of 4, suggesting the absence of deep pockets (13).

The health of periodontal tissue can be seen from various examinations such as measurements investigating depth, measurement of clinical attachment loss, and bleeding on probing (BoP) bleeding examination. If BoP presents it is an early sign of gingivitis and precedes visual signs of clinical inflammation such as swelling and redness. BoP can be assessed by pressure from the periodontal probe. Probing pressure to apply to periodontal pocket tissue when performing BoP should not cause trauma, but only be sufficient to trigger tissue bleeding if there is fragility of blood vessels due to inflammation (14).

Bleeding when probing gently indicates objective inflammation parameters, which have been incorporated into known index systems, for periodontal evaluation. The percentage of teeth with bop can also be used as an individual assessment parameter representing the response of periodontal tissue to the bacterial challenge. Incorporation of BoP percentage into pre-established a BoP prevalence of 25% as the borderline point between patients maintaining periodontal stability for 4 years and patients with recurrent disease within the same time frame (15).

Building upon this foundation, the present study titled “The Relationship of Stress Levels with Periodontal Tissue Health in Soldiers from Kodam Jaya aims to assess the oral and periodontal health status of soldiers, particularly in relation to stress levels, within the context of Kodam Jaya Soldier.

## 2 Methods

This cross-sectional study explores the relationship between stress levels and periodontal tissue health among soldiers from Kodam Jaya. The sample size was determined using the Harry King Nomogram technique, which indicated a minimum requirement of 43 participants. After applying dropout criteria, the final sample consisted of 48 male respondents aged between 18 and 35 years. A purposive sampling method was employed for this study. The inclusion criteria included soldiers from Kodam Jaya who were willing to participate and had periodontal tissue disorders. Conversely, the exclusion criteria included individuals who were not soldiers of Kodam Jaya and those who declined to participate in the research. The study was conducted at Kodam Jaya, Rawa Lumbu, Bekasi, in January 2024.

A closed questionnaire consisting of ten questions regarding feelings and thoughts from the previous month, along with the Perceived Stress Scale (PSS), will be utilized to gather primary data aimed at exploring the relationship between stress levels and the health of periodontal tissue. Informed consent was obtained before participation. Statistical analyses were conducted using SPSS (Statistical Product and Service Solutions). Univariate analysis was applied to illustrate the characteristics of each variable, presented as frequency distributions and percentages or proportions. Bivariate analysis was employed to assess the relationship between stress levels and periodontal tissue health among soldiers from Kodam Jaya. Additionally, a Spearman-rho correlation test was performed to evaluate the condition of periodontal tissue during the BoP examination.

## 3 Results

The study was conducted in February 2024 at the Kodam Jaya to determine the Relationship between Stress Level and Periodontal Tissue Health in Soldiers from Kodam Jaya. A total of 48 soldiers have agreed and signed informed consent as a condition of participating in the study. The results of the analysis that will be presented in this study are as follows.

### 3.1 Univariate analysis

**Figure 1:**
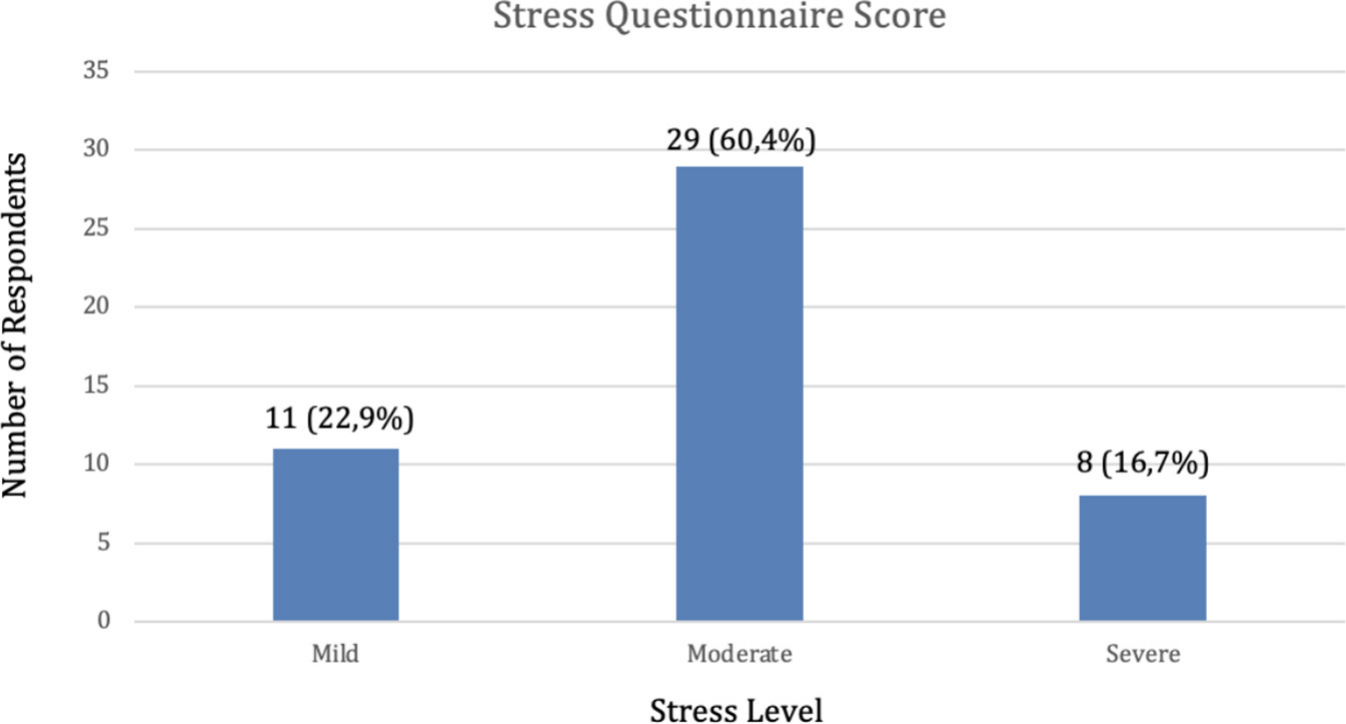
Frequency distribution of respondents’ stress questionnaire score.

This univariate analysis is to see the frequency distribution of stress level criteria and periodontal tissue health with Bleeding On Probing (BoP) examination. The results of Univariate Analysis in this study can be seen from the following table:

The frequency distribution of respondents’ stress questionnaire scores is shown in the following table:

Table 1 shows that out of 48 respondents, it can be seen that the stress questionnaire scores of respondents in the mild category were 11 respondents (22.9%), the moderate category were 29 respondents (60.4%), and the severe category were 8 respondents (16.7%) (Figure 1).

**Table 1:** Frequency distribution of respondents’ stress questionnaire scores.

Criteria	f	%
Mild	11	22.9%
Moderate	29	60.4%
Severe	8	16.7%
Total	48	100%

**Figure 2:**
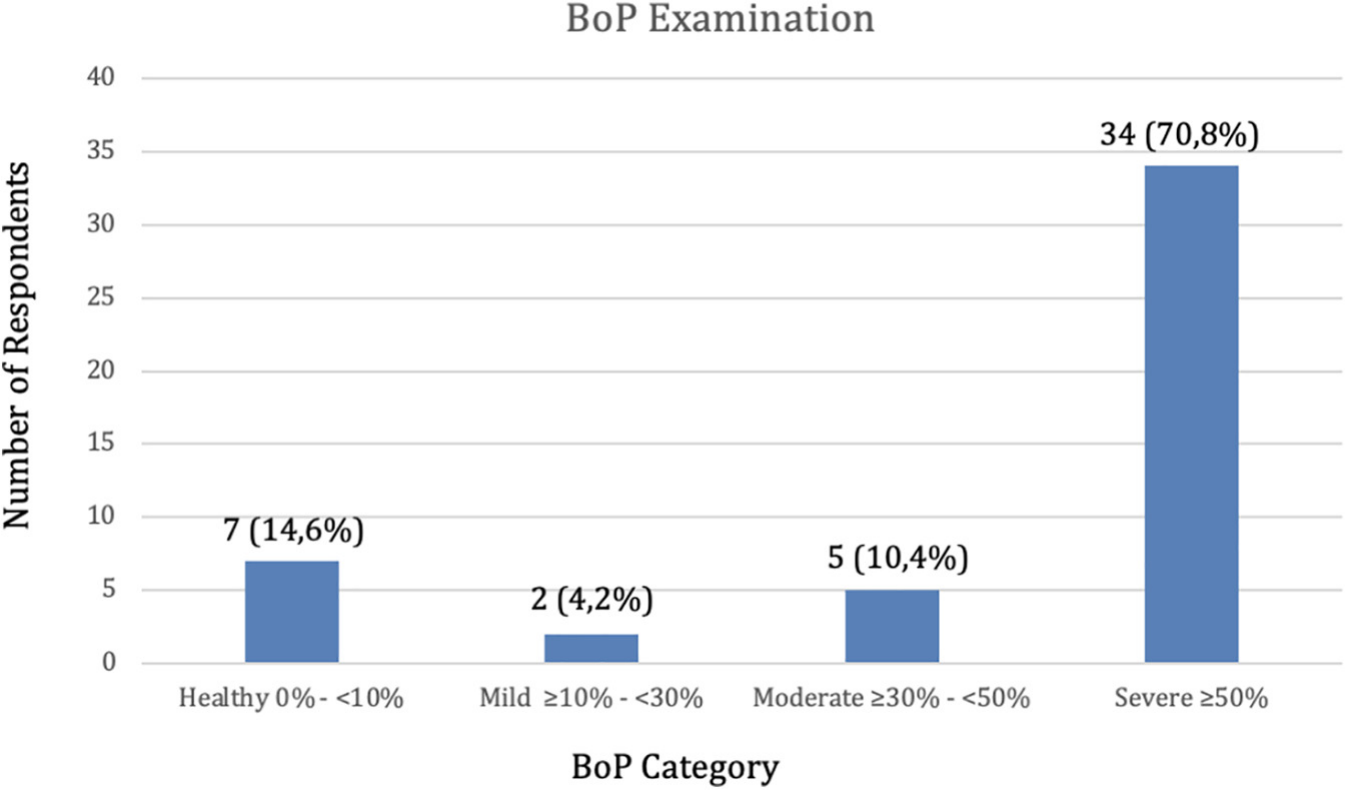
Frequency distribution of BoP examination.

In Table 2, the frequency distribution of BoP examination in healthy criteria is 7 respondents (14.6), mild criteria are 2 respondents (4.2), moderate criteria are 5 respondents (10.4), severe criteria are 34 respondents (70.8) (Figure 2).

**Table 2:** Frequency distribution of BoP inspection.

Criteria	f	%
Healthy 0% -<10%	7	14.6
Mild ≥10% -<30%	2	4.2
Moderate ≥30% -<50%	5	10.4
Severe ≥50%	34	70.8
Total	48	100%

### 3.2 Bivariate analysis

Bivariate analysis in this study used the Spearman-Rho correlation test to determine the relationship between stress levels and periodontal tissue health. If the resulting *p* value ≤ *α* 0.05 indicates there is a relationship between the two variables, and if the *p* value > *α* 0.05 indicates there is no relationship between the two variables. The direction of the relationship between the two variables is shown by the value of the rho coefficient (r). A positive r value indicates a unidirectional relationship, while a negative r value indicates a unidirectional relationship. The strength of the relationship is classified based on the rho (r) correlation value according to Sugiyono (16).

Based on Table 3, it shows that there is a relationship between stress levels and periodontal tissue health on BoP examination (*p*-value = 0.004) with a moderate level of strength of relationship and a positive direction of relationship (r = 0.407).

**Table 3:** Correlation between stress level and BoP measurement.

Variable	*p*-value	r
BoP categoric	0.004	0.407*

*Spearman-rho correlation.

## 4 Discussion

The aforementioned data was collected in the Kodam Jaya in February 2024, with a sample size of 48 participants. The research data was subsequently analyzed using a computer statistical programme, such as SPSS, to determine if there was a correlation between stress levels and the health of periodontal tissue during the Bleeding on Probing (BoP) examination.

Univariate analysis is employed to elucidate the nature of each individual variable. The frequency distribution analysis of stress levels among soldiers reveals that the light category comprises 11 individuals (22.9%), the moderate category consists of 29 individuals (60.4%), and the heavy category includes 8 individuals (16.7%) (Figure 1). Therefore, out of the total 48 responses, the moderate stress level is the most prevalent among Battalion Soldiers. Regarding the frequency distribution of BoP examinations, the healthy criteria accounted for 7 cases (14.6%), mild criteria for 2 cases (4.2%), moderate criteria for 5 cases (10.4%), and severe criteria for 34 cases (70.8%) (Figure 2). Additionally, high criteria were observed. The most prevalent BoP examination occurs during the examination of respondents.

The research findings indicate a significant relationship between stress levels and periodontal tissue health based on the BoP examination results in the BoP category. High levels of stress can have a substantial impact on periodontal tissue health. Psychological stress can trigger immunological changes that lead to an inflammatory response, tissue damage, and attachment loss in periodontal tissue. Stress hormones such as CRH, ACTH, and glucocorticoids can influence the immune response, resulting in bone and periodontal tissue damage. Studies also show that the bacterium *Porphyromonas gingivalis*, commonly found in dental plaque, can induce pathological changes in periodontal tissue by activating the host's immune and inflammatory responses, directly affecting periodontal cells. Therefore, effective stress management, coupled with proper oral healthcare, is crucial to prevent more severe periodontal conditions.

Stress has been increasingly recognized as a significant factor influencing periodontal health. The relationship between psychological stress and periodontal disease is complex, involving both direct biological mechanisms and indirect behavioral pathways. This discussion explores the multifaceted connection between stress levels and periodontal tissue health, drawing on recent studies published in high-impact medical journals.

### 4.1 Biological mechanisms linking stress and periodontal disease

Psychological stress can adversely affect periodontal health through various biological pathways. Activation of the hypothalamic-pituitary-adrenal (HPA) axis during stress leads to increased secretion of cortisol, a glucocorticoid that modulates immune function. It is produced by the adrenal gland and can raise blood glucose levels and weaken the immune system, making a person more vulnerable to periodontal disease. Elevated cortisol levels in blood serum and saliva are frequently seen in people with periodontitis and high stress levels. Elevated cortisol levels can suppress the immune response, reducing the body's ability to combat periodontal pathogens effectively. This immunosuppression facilitates bacterial colonization and exacerbates inflammatory processes within periodontal tissues. When a person experiences stress, their body produces corticotropin-releasing hormone (CRH), which can stimulate gingival mast cells. This, in turn, can cause the mast cells to release pro-inflammatory molecules along with other neuropeptides and cytokines, ultimately leading to periodontitis. Additionally, stress-induced alterations in cytokine profiles, including increased production of pro-inflammatory cytokines like interleukin-6 (IL-6) and tumor necrosis factor-alpha (TNF-α), contribute to periodontal tissue breakdown. These cytokines promote the recruitment of inflammatory cells and the release of matrix metalloproteinases (MMPs), enzymes responsible for the degradation of extracellular matrix components, leading to connective tissue destruction and alveolar bone resorption.

Although previous literature suggests that gender differences may influence stress and periodontitis, this study did not assess such differences, as all participants were male. The gender-based discussion is included solely for contextual purposes. In addition to professional responsibilities, women frequently have an overabundance of cultural expectations, including those related to their personal lives, bodies, hormones, sexuality, and society. Conversely, males are more likely to have periodontitis, and some research indicates that sex hormones—specifically, elevated testosterone levels—may be linked to the condition.

### 4.2 Behavioral factors mediating the stress-periodontal disease connection

Beyond physiological mechanisms, stress influences behaviors that can indirectly impact periodontal health. Individuals experiencing high stress levels may adopt detrimental habits such as neglecting oral hygiene, increasing tobacco use, and altering dietary patterns, all of which are risk factors for periodontal disease. Stress-related behaviors, including bruxism (teeth grinding) and clenching, can cause mechanical trauma to periodontal tissues, further exacerbating periodontal conditions. Moreover, stress can lead to decreased salivary flow, resulting in xerostomia (dry mouth), which compromises the protective functions of saliva, such as buffering capacity and antimicrobial activity, thereby creating a more conducive environment for periodontal pathogens. Additionally, medications that induce xerostomia (dry mouth), like certain antidepressants or antihistamines, reduce saliva flow, which can promote plaque accumulation and increase the risk of periodontal disease.

### 4.3 Evidence from recent clinical studies

Recent studies have provided further insights into the association between stress and periodontal disease. A 2024 cross-sectional study by Macri et al. assessed the relationship between perceived stress, mindfulness, and periodontal health among 203 participants. The study found that higher perceived stress levels were significantly associated with poorer periodontal health, as indicated by increased Periodontal Screening and Recording (PSR) scores and Gingival Bleeding Index (GBI) values. Conversely, higher mindfulness scores were associated with better periodontal health outcomes, suggesting that mindfulness may mitigate the adverse effects of stress on periodontal tissues (17).

Another study published in 2022 by Zhang et al. explored the neuroimmune mechanisms underlying the impact of psychological stress on periodontal disease. The researchers highlighted that stress-induced alterations in the neuroimmune system could disrupt periodontal homeostasis, leading to increased susceptibility to periodontal inflammation and tissue destruction. The study emphasized the need for further research to elucidate the complex interactions between psychological stress, neuroimmune responses, and periodontal health (18).

### 4.4 Implications for clinical practice

Understanding the relationship between stress and periodontal health has significant implications for clinical practice. Periodontal treatment plans should incorporate assessments of patients’ stress levels and related behaviors. Interventions aimed at stress reduction, such as cognitive-behavioral therapy, relaxation techniques, and lifestyle modifications, may enhance periodontal treatment outcomes. Additionally, educating patients about the impact of stress on oral health and promoting effective stress management strategies can serve as preventive measures against periodontal disease. Clinicians should adopt a holistic approach, considering both physiological and psychological factors, to provide comprehensive periodontal care.

### 4.5 Limitations of the study

This study has several limitations that should be acknowledged. First, as a cross-sectional study, it inherently lacks the ability to establish causal relationships; it can only provide supportive evidence for the proposed hypothesis. Second, the relatively small sample size and the inclusion of only male participants limit the generalizability of the findings to broader populations, including female military personnel. Third, the study did not assess biological mediators of stress, such as cortisol levels in saliva or serum, which could have offered more objective physiological evidence of the respondents’ stress levels.

### 4.6 Future research directions

While existing studies have established a link between stress and periodontal disease, further research is needed to elucidate the underlying mechanisms and to develop targeted interventions. Future studies should explore the role of specific stress-related biomarkers in periodontal disease progression and assess the efficacy of stress management programs in improving periodontal outcomes. Additionally, investigating the interplay between stress, genetic predisposition, and environmental factors could provide a more comprehensive understanding of individual susceptibility to periodontal disease.

## 5 Conclusion

Based on the researchers’ findings and analysis, it can be inferred that there is a significant correlation between stress and the health of periodontal tissue, as well as the BoP examination, among Soldiers from Kodam Jaya.

## Data Availability

The raw data supporting the conclusions of this article will be made available by the authors, without undue reservation.

## References

[1] Mossie A Kindu D Negash A. Prevalence and severity of depression and its association with substance use in Jimma town, southwest Ethiopia. Depress Res Treat. (2016) 2016:3460462. 10.1155/2016/3460462 27069680 PMC4812317

[2] Aisa T Diviney D Thomas J Al Qadheeb N Abdelbaky M Afify H Stress level assessment among health care workers involved in the management of critically ill COVID-19 patients. Ir J Med Sci. (2022) 191(3):1067–73. 10.1007/s11845-021-02721-0 34333738 PMC8325526

[3] Yolanti RI Sitam S Wandawa G Pramanik F. Estimasi usia prajurit tni al berdasarkan tooth coronal index pada digital radiograf panoramik. Jurnal PDGI. (2020) 2020(3):61–6. 10.32793/jrdi.v4i3.591

[4] Lastovkova A Carder M Rasmussen HM Sjoberg L Groene GJ Sauni R Burnout syndrome as an occupational disease in the European union: an exploratory study. Ind Health. (2018) 56(3):254–63. 10.2486/indhealth.2017-0132 PMC5889935 29109358

[5] Soumena R Husada SM. Faktor-faktor yang berhubungan dengan stres kerja pada perawat diruang rawat inap rumah sakit umum daerah namrole. Jurnal Keperawatan. (2021) 14(2):132. 10.30598/molmed.2021.v14.i2.132

[6] Wisén N Larsson G Risling M Arborelius U. Measuring the impact of operational stress: the relevance of assessing stress-related health across the deployment cycle. Mil Med. (2023) 188(7–8):e2126–132. 10.1093/milmed/usab542 34977944 PMC10363009

[7] Vasiliou A Shankardass K Nisenbaum R Quiñonez C. Current stress and poor oral health. BMC Oral Health. (2016) 16:94. 10.1186/s12903-016-0284-y 27590184 PMC5010733

[8] Isola G Santonocito S Lupi SM Polizzi A Sclafani R Patini R Periodontal health and disease in the context of systemic diseases. Mediat Inflamm. (2023) 2023:9720947. 10.1155/2023/9720947 PMC10199803 37214190

[9] Madiba T Bhayat A. Periodontal disease - risk factors and treatment options. South African Dental Journal. (2018) 73(9):571–5. 10.17159/2519-0105/2018/v73no9a5

[10] Cho YD Kim KH Lee YM Ku Y Seol YJ. Periodontal wound healing and tissue regeneration: a narrative review. Pharmaceuticals. (2021) 14(5):456. 10.3390/ph14050456 34065862 PMC8151433

[11] Castro MML Duarte NN Nascimento PC Magno MB Fagundes NCF Flores-Mir C Antioxidants as adjuvants in periodontitis treatment: a systematic review and meta-analysis. Oxid Med Cell Longevity. (2019) 2019:9187978. 10.1155/2019/9187978 PMC6679881 31428231

[12] Dakovic D Lekic M Bokonjić D Lazic Z Cutovic T Mladenović R. Evaluation of periodontal status and treatment needs of the Serbian military forces population. Vojnosanit Pregl. (2020) 78:10–10. 10.2298/VSP191125010D

[13] Setyawati BP Agis Marludia M Puspitawati Y Nabilah Sari S Nurwanti W Kesehatan Gigi Ditkesad A. Pemeriksaan dan edukasi kesehatan jaringan periodontal pada prajurit dikjurtakes abit dikmata tni ad. Gemakes. (2022) 2(1):19–23. 10.36082/gemakes.v2i1.532

[14] Barbosa VL Angst PD Finger Stadler A Oppermann RV Gomes SC. Clinical attachment loss: estimation by direct and indirect methods. Int Dent J. (2016) 66:144–9. 10.1111/idj.12218 26846817 PMC9376648

[15] Heitz-Mayfield LJA Heitz F Lang NP. Implant disease risk assessment idra–a tool for preventing peri-implant disease. Clin Oral Implants Res. (2020) 31(4):397–403. 10.1111/clr.13585 32003037

[16] Sugiyono. Metode Penelitian Kombinasi (Mixed Methods). Bandung: CV Alfabeta (2018).

[17] Macrì M D’Albis G D’Albis V Antonacci A Abbinante A Stefanelli R Periodontal health and its relationship with psychological stress: a cross-sectional study. J Clin Med. (2024) 13(10):2942. 10.3390/jcm13102942 38792482 PMC11122378

[18] Zhang J Lin S Luo L Zhang Q Jiao Y Liu W. Psychological stress: neuroimmune roles in periodontal disease. Odontology. (2022) 110(3):455–64. 10.1007/s10266-022-00768-8 36437431

